# Collaborative health research partnerships: a survey of researcher and knowledge-user attitudes and perceptions

**DOI:** 10.1186/s12961-019-0485-3

**Published:** 2019-11-27

**Authors:** Shannon L. Sibbald, Hosung Kang, Ian D. Graham

**Affiliations:** 1 University of Western Ontario, 1151 Richmond St, London, ON N6A 3K7 Canada; 2 University of Ottawa, 600 Peter Morand Crescent, Ottawa, ON K1G 5Z3 Canada

**Keywords:** Integrated knowledge translation, funded research, grants, partnerships

## Abstract

**Background:**

Integrated knowledge translation describes the process of partnered research between different stakeholders with the goal of producing research that ultimately achieves a greater impact when put into practice. A better understanding of research partnerships and integrated knowledge translation has implications for future partnerships and collaborative initiatives in practice. Our research describes and expands upon previous work done to identify barriers and attitudes toward collaboration in the context of research funding opportunities that required researcher–knowledge-user partnerships.

**Methods:**

A survey was sent out to researchers funded by the Canadian Institutes of Health Research and knowledge-users who worked collaboratively on their research projects. There were two mirror versions of the survey, one for researchers and one for knowledge-users. Descriptive statistics, χ^2^ analysis and Mann–Whitney U analysis were used to understand the processes, barriers, perceived impact and sustainability of the partnerships.

**Results:**

The results revealed that, although there were differences in the roles of researchers and knowledge-users, both groups felt very positive towards their partnerships. Some of the barriers identified as inhibiting effective partnerships were resource constraints (funding/time) and differences in contribution and involvement amongst team members. Despite these barriers, both researchers and knowledge-users felt that the partnership was not only sustainable, but also helped create an impact.

**Conclusions:**

Our results provide useful information for funding agencies launching opportunities requiring or encouraging collaborative research projects between researchers and knowledge-users.

## Background

Over the past 15–20 years in Canada, and elsewhere, there has been a shift towards forming research partnerships to enhance the relevance and impact of research findings and facilitate their application in the real world. Research funders in Canada [1], Australia [2], the United Kingdom [3], the Netherlands [4], and the United States [5] have created partnership-based funding opportunities. Partnered research between researchers and those who would benefit from the knowledge gained by their research (i.e. all types of decision-makers, including patients, clinicians, health system managers, policy-makers, etc.) has been proposed and studied as a means to shape research questions efficiently as well as to increase research uptake and impact [6–9]. Engaging both researchers and knowledge-users in research is a complex task that involves the integration of individuals with various skillsets to conduct and interpret the results for practical application [10]. The Canadian Institutes of Health Research (CIHR), Canada’s premier health research funding agency, calls this approach to research, that partners researchers and knowledge-users, ‘integrated knowledge translation’ (IKT) [11]. Regardless of the name, most agree that researcher–knowledge-user partnerships are complex relationships that involve a great deal of coordination and collaboration [12].

In the context of researcher and knowledge-user partnerships, IKT manifests itself in various ways, including forming research questions, interpreting findings and applying findings in practice [13]. When IKT is performed well, it has the potential to optimise healthcare delivery systems and improve health system performance through its various mechanisms that promote collaboration, dynamic research and engagement among different actors [14, 15]. Several studies reporting on IKT mechanisms suggest that IKT can support the uptake of research into policy through collaboration (inherent in IKT), improved communication, trust and a shared vision [16]. IKT has also been shown to help inform research questions relevant to practice and policy as well as help with interpreting findings based on contextual knowledge [17]. Other studies have reported less favourable outcomes during IKT, such as lack of funding or incentives, failure to overcome differences, and little to no research produced [16]. More research is required to better understand how different mechanisms of IKT function in different contexts and which mechanisms achieve the best results [13, 18].

The CIHR used targeted IKT funding opportunities as a mechanism to encourage and support researcher and knowledge-user collaborations with the intent of generating and applying the acquired knowledge to increase the impact of research funding (i.e. address societal health issues). These CIHR funding opportunities included Partnerships for Health System Improvement (PHSI), Knowledge Synthesis (KS) and Knowledge to Action (KTA) grants. PHSI was primarily a 3-year operating grant used to support researchers and decision-makers (health system policy-makers and/or managers) in the development of applied health services research intended to improve the efficiency and effectiveness of the healthcare system. KS grants aimed to support scoping reviews and knowledge syntheses that described the current state of knowledge on a topic to inform knowledge-users of the lack/existence of evidence to support their decision-making process and guide future research. Lastly, KTA grants aimed to improve research application or uptake by knowledge-users and evaluate how effectively this was achieved. The funding provided for PHSI grants included $600,000 CAD over the course of 3 years and applicants had to secure a minimum of 20% of the grant awarded by CIHR from other partners. Funding for KS included $100,000 CAD for up to 1 year for a knowledge synthesis and $50,000 CAD for up to 1 year for a scoping review. KTA grants were 2 years in length for a maximum of $200,000 CAD. A funding requirement for all three opportunities was that knowledge-users had to be included as named co-applicants on the grant proposals and, in the case of the PHSI grant opportunity, a decision-maker had to be listed as a principal co-applicant. Letters of support from knowledge-user co-applicants were used by the merit review panel to assess the nature and extent of the researcher–knowledge-user partnership. Merit review was used to adjudicate these grant proposals and differed from more traditional peer-review in that the reviewers included approximately equal numbers of researchers and knowledge-users, both of whom evaluated the proposals’ potential scientific merit, relevance and impact [19]. Proposals had to score high on all criteria to be funded.

Between 2010 and 2012, the knowledge translation (KT) portfolio at CIHR conducted a mixed methods study of successful principal researchers and knowledge-users of PHSI, KS and KTA grants funded between 2005 and 2009 as part of a quality improvement initiative [20]. The study involved conducting an online survey followed by qualitative interviews with a subsample of the survey respondents. The results of the qualitative study have been reported elsewhere [20] and revealed the benefits of partnered research as it relates to shaping research questions, the research process itself and applying the research outcomes in practice. Additionally, it described the potential for funding agencies to support and foster partnership development in research. The qualitative study revealed that the respondents did not consider there to be one best approach for partnership success. Instead, participants identified four key factors that increased their likelihood of success – (1) a partnership built on an existing relationship, (2) alignment of researcher and knowledge-user agendas, (3) having a skilled researcher involved in the grant, and (4) regular, multi-modal research team communication.

The survey findings were used to inform the design of CIHR’s subsequent evaluation of its KT funding programme [21]; however, a comprehensive analysis of the findings was never undertaken, reported or published. We believe this study remains relevant today as a historical baseline from the middle of the first decade of the 21st century. This survey was the first attempt to systematically understand researcher–knowledge-user experiences and impact from a large cohort of IKT grants funded by a national health research funding agency. In this paper, we analyse and report on the survey findings to identify how researcher–knowledge-user partnerships were perceived, barriers that were identified by both parties, factors that led to successful partnerships in the future and how these partnerships can impact research uptake.

### Methods

It is difficult to directly observe and measure researcher–knowledge-user collaborations; therefore, surveys were used to try and understand the beliefs and attitudes associated with this phenomenon [22]. Items in the survey were created based on a literature review focused on researcher–knowledge-user partnerships commissioned by CIHR [23]. Ten dominant barriers to successful partnerships were identified in the literature review, which informed the eight areas of inquiry addressed in the survey; these were details of the partnership, design of the study, outcomes, required partnerships, processes, knowledge sharing, next steps, and factors facilitating partnerships. There were two mirror versions of the survey, one for the researchers and one for the knowledge-users (copies of each survey are available upon request), each with 41 questions seeking to understand how the research process benefitted from the partnership and the perceptions and interpretations of the respective study results from both groups. A 5-point Likert scale as well as ranking and open-ended questions were used. The survey was piloted to ensure face and content validity. The survey was posted online and the full Dillman methodology was used to encourage researchers and knowledge-users to respond [24]. Furthermore, the reporting guideline ‘Good practice in the conduct and reporting of survey research’ [25] was used throughout the research.

A pre-notification email was sent to all grant holders using a CIHR database as the sampling frame. A link to the survey was sent out using the same email list; participants were also sent three follow-up/reminder emails.

The specific research questions guiding the analysis of the survey data were (1) what was the partnership process like and how was it perceived among both groups (how are knowledge-users engaged in the research process), (2) what kinds of barriers were experienced in the partnership, (3) what was the perceived impact of the partnership, and (4) was the relationship perceived to be sustainable? The type of respondent (researcher or knowledge-user) was used to interrogate the findings.

All data analyses were done using Statistical Package for the Social Sciences (SPSS, version 25.0). Analysis of the survey data included examining simple descriptive statistics and frequency distributions pertaining to the proportional responses provided by researchers and knowledge-users. Additional non-parametric tests, such as χ^2^ tests and Mann–Whitney U tests, were performed to compare the responses of knowledge-users and researchers since the scores were ordinal. When using Mann–Whitney U tests, the responses of respondents who stated ‘too early to tell’ were removed from the analysis to maintain the nature of the ordinal scale measurements. The number of participants varied throughout the results due to not everyone responding to every question and depending on the type of analysis. Significance was established if the *P* value was less than 0.05 and all analyses involved two-tailed tests.

## Results

The online survey was sent to 174 principal researcher applicants and 106 knowledge-user applicants, of whom 141 researcher applicants and 75 knowledge-user applicants completed the survey, giving response rates of 81% and 71%, respectively. The majority of both researchers (*n* = 101, 72.1%) and knowledge-users (*n* = 51, 75%) reported that the study was not finished at the time the survey was filled out. In general, the most common knowledge-users for PHSI grants are health systems managers, policy-makers, healthcare providers and, less commonly, patients. For KS grants, knowledge-users were most often clinicians. Lastly, for KTA grants knowledge-users varied.

### The partnering process

Researchers and knowledge-users were asked to report if they developed a process or strategy to handle items such as ongoing communication about the study, information technology, coordination of work plan and deliverables, conflict management, development and authorship of papers, and dissemination of findings. Most researchers (*n* = 110, 78%) and knowledge-users (*n* = 49, 65%) had discussed these items prior to or during the study. When asked the same question about conflict management strategies, 92 (68.7%) researchers and 46 (71.9%) knowledge-users reported that a strategy was never discussed.

A χ^2^ test of independence revealed that there were significant differences between researchers and knowledge-users about their perceived roles (χ^2^ (5, *N* = 200) = 132.59, *P* ≤0.001). Most researchers believed that they were more in a lead role (*n* = 101, 78.3%), while most knowledge-users reported taking on more of an advisory role (*n* = 34, 47.9%). Knowledge-users rarely reported taking the lead on projects. Despite the differences in opinion, the majority of researchers and knowledge-users reported that most of the project (from beginning to finish) was done collaboratively (Table 1). Researchers and knowledge-users agreed that researchers had more of a lead role during the initial research phases (e.g. shaping the research question, deciding on methodology, and data collection and tool development) and less during the later stages (such as interpreting findings, moving the research results into practice, and their widespread dissemination and application).

**Table 1:** Researcher and Knowledge-users involvement in different stages of the project

	Researcher responses				Knowledge-user responses			
	Knowledge-user(s) leading	Collaboration	Researcher leading	Total	Knowledge-user(s) leading	Collaboration	Researcher leading	Total
Shaping the research question(s)	1 (0.7%)	124 (87.9%)	16 (11.3%)	141	1 (1.4%)	58 (79.5%)	14 (19.2%)	73
Deciding on the methodology	0	87 (61.7%)	54 (38.3%)	141	0	50 (68.5%)	23 (31.5%)	73
Data collection and tools development	0	94 (67.6%)	45 (32.4%)	139	0	52 (71.2%)	21 (28.8%)	73
Interpreting findings and crafting messages	1 (0.8%)	110 (91.7%)	9 (7.5%)	120	0	54 (88.5%)	7 (11.5%)	61
Moving the research results into practice	3 (2.7%)	104 (92.0%)	6 (5.3%)	113	1 (1.7%)	53 (91.4%)	4 (6.9%)	58
Widespread dissemination and application	0	108 (96.4%)	4 (3.6%)	112	0	53 (93%)	4 (7.0%)	57

When asked how the partnership shaped the way they thought about research, researchers reported that it changed the way they think about research design significantly more than knowledge-users (*U* = 1981, *P* ≤0.001, *r* = − 0.48; mean rank of knowledge-user and researcher were 64.14 and 125.11, respectively). Conversely, knowledge-users reported that their thinking around research had not been changed as a result of the partnership.

The survey asked about access to knowledge and information as a key function of the partnership. Researchers (*n* = 68, 46.5%) and knowledge-users (*n* = 36, 48.6%) felt that their partnership broadened access to different forms of knowledge. Some researchers (*n* = 25, 18.2%) and knowledge-users (*n* = 12, 16.2%) reported that the partnership did little or nothing to broaden access to knowledge and information.

There were differences between researchers and knowledge-users as to when trust was established within the partnership. A χ^2^ test of independence revealed that there was a significant difference in the responses of researchers and knowledge-users (χ^2^ (3, *N* = 192) = 18.75, *P ≤*0.001). Researchers (*n* = 83, 66.9%) reported that it took months or longer to develop trust, whereas knowledge-users (*n* = 46, 61.3%) reported that it took weeks. Both groups reported positively about the level of communication between partners throughout the project. Most researchers (*n* = 130, 87.8%) and knowledge-users (*n* = 73, 90%) reported that the level of communication was satisfactory (score of 4) or greater (score of 5) on the 5-point Likert scale. Knowledge-users reported significantly greater satisfaction with the quality of communication than researchers (*U* = 4257, *P* = 0.031, *r* = 0.14). However, this does not indicate that researchers were dissatisfied with the quality of communication in their partnerships.

### Partnership barriers

When asked about the barriers experienced in the partnership, the most prominent factors reported by both groups were ‘inadequate resources’ followed by ‘concern about the quality of the research’ and ‘incompatibility of problem-solving styles’ (Tables 2 and 3). Almost 85% of researchers (*n* = 111) reported that adequate resources (money and personnel) affected their ability to complete their study (from a little to a significant amount). Similarly, almost 69% of knowledge-users (*n* = 46) reported that ‘inadequate resources’ affected their ability to complete this study. Researchers rated ‘inadequate resources’ as affecting them significantly more than knowledge-users (*U* = 2972, *P* <0.001, *r* = − 0.26). Whereas 50% of researchers (*n* = 65) reported that concerns about the quality of the research affected their ability to complete the study, 78.8% of knowledge-users (*n* = 52) reported that the same factor did not affect their ability to complete the study. In contrast, 73.3% of researchers (*n* = 96) and 55.4% of knowledge-users (*n* = 36) reported that ‘differences in contribution amongst team members’ affected their ability to complete the study.

**Table 2:** Barriers experienced in the partnership – researcher responses

Researcher	Not at all	A little	Somewhat	A lot	A significant amount	Total
Adequate resources (money and personnel)^a^	20 (15.3%)	20 (15.3%)	33 (25.2%)	31 (23.7%)	27 (20.6%)	131
Concerns about the quality of the research^a^	64 (49.6%)	18 (14%)	23 (17.8%)	12 (9.3%)	12 (9.3%)	129
Compatibility of problem-solving styles amongst team members^a^	61 (46.6%)	28 (21.4%)	23 (17.6%)	16 (12.2%)	3 (2.3%)	131
Level of trust amongst team members	72 (55.4%)	18 (13.8%)	16 (12.3%)	15 (11.5%)	9 (6.9%)	130
Amount of turnover amongst team members^a^	65 (49.2%)	20 (15.2%)	30 (22.7%)	6 (4.5%)	11 (8.3%)	132
Power/status imbalances amongst team members	88 (68.2%)	23 (17.8%)	14 (10.9%)	3 (2.3%)	1 (0.8%)	129
Knowledge/skill imbalances amongst team members	78 (59.5%)	29 (22.1%)	16 (12.2%)	5 (3.8%)	3 (2.3%)	131
Competing agendas amongst team members^a^	57 (43.8%)	34 (26.2%)	20 (15.4%)	16 (12.3%)	3 (2.3%)	130
Differences in availability/contribution amongst team members^a^	35 (26.7%)	41 (31.3%)	35 (26.7%)	15 (11.5%)	5 (3.8%)	131
Lack of financial or professional incentives for conducting this type of research for the researcher(s) on the team	81 (62.3%)	20 (15.4%)	22 (16.9%)	5 (3.8%)	2 (1.5%)	130
Lack of financial or professional incentives for conducting this type of research for the knowledge-user(s) on the team^a^	52 (40.6%)	36 (28.1%)	23 (18%)	15 (11.7%)	2 (1.6%)	128

^a^Indicates where more than 50% of the group responded to have at least ‘a little’ impact to barriers

**Table 3:** Barriers experienced in the partnership – knowledge-user responses

Knowledge-user	Not at all	A little	Somewhat	A lot	A significant amount	Total
Adequate resources (money and personnel)^a^	21 (31.3%)	16 (23.9%)	18 (48.6%)	5 (13.5%)	7 (18.9%)	67
Concerns about the quality of the research	52 (78.8%)	6 (9.1%)	6 (9.1%)	2 (3%)	0	66
Compatibility of problem-solving styles amongst team members	41 (62.1%)	18 (27.3%)	4 (7.1%)	2 (3.6%)	1 (1.5%)	66
Level of trust amongst team members	49 (74.2%)	7 (10.6%)	4 (6.1%)	4 (6.1%)	2 (3%)	66
Amount of turnover amongst team members	54 (81.8%)	8 (12.1%)	4 (6.1%)	0	0	66
Power/status imbalances amongst team members	51 (77.3%)	8 (12.1%)	4 (6.1%)	0	3 (4.5%)	66
Knowledge/skill imbalances amongst team members	43 (66.2%)	14 (21.5%)	7 (10.8%)	1 (1.5%)	0	65
Competing agendas amongst team members	40 (62.5%)	14 (21.9%)	7 (10.9%)	3 (4.7%)	0	64
Differences in availability/contribution amongst team members^a^	29 (44.6%)	25 (38.5%)	6 (10.9%)	2 (3.1%)	3 (4.6%)	65
Lack of financial or professional incentives for conducting this type of research for the researcher(s) on the team	44 (67.7%)	16 (29.1%)	3 (4.6%)	1 (1.5%)	1 (1.5%)	65
Lack of financial or professional incentives for conducting this type of research for the knowledge-user(s) on the team	38 (58.5%)	19 (29.2%)	7 (10.8%)	1 (1.5%)	0	65

^a^Indicates where more than 50% of the group responded to have at least ‘a little’ impact to barriers

### Research outputs and perceived impact

While most respondents reported that the study was not finished at the time the survey was completed, many researchers and knowledge-users agreed that their research project has had, or would have, an overall impact. For some, this was best described through research outputs, such as published abstracts, manuscripts in press, and “*high quality publications*” (researcher). Both researchers and knowledge-users reported the creation of manuscripts (or articles), other published works and general dissemination of their findings as tangible project outputs.

Even though researchers and knowledge-users reported that it was still too early to see any impact, many participants acknowledged that the work they completed had a tangible impact on policy and/or practice as a direct result of incorporating the partnership into the research process. Most researchers (*n* = 88, 85.4%) and knowledge-users (*n* = 44, 73.3%) believed that being in a partnership increased the uptake of the study results. For example, researchers described that uptake of deliverables by target populations improved due to better and more comprehensive consultations with stakeholders. The majority of both researchers (*n* = 117, 86.7%) and knowledge-users (*n* = 59, 80.8%) believed their study was more likely to have an impact compared to other studies that did not involve researcher/knowledge-user partnerships. Knowledge-users provided examples of making more robust changes to practice as a result of their involvement in the partnership. Researchers believed that the grant provided necessary background information for subsequent grants, allowing for the development of new phases of projects as well as further understanding and awareness of their research.

For many researchers and knowledge-users, the impact was more about laying the groundwork for future research, “*validat*[ing] *the gap in the evidence and where we should focus our implementation and evaluation strategies*” (knowledge-user), and “*provid*[ing] *preliminary data*” (researcher) for future studies. There were a few open-ended comments that pointed to a less favourable view of how the partnership impacted the research, such as “[the] *impact* [was] *seriously blunted by changes in the health care environment*” (researcher). Some knowledge-users were “*unaware of the impact of the grant*” or reported it having no direct impact on their organisation. Overall, the majority of both researchers (*n* = 118, 89.7%) and knowledge-users (*n* = 64, 90.1%) perceived their partnership as effective (defined as mutually beneficial and productive).

Open-ended feedback regarding optimising future partnerships reinforced that requiring partnerships has the potential to positively influence research outcomes as well as the overall impact of the grant; one researcher commented that all grants (in particular those funded through federal agencies such as CIHR) should involve required partnerships. This was echoed by a knowledge-user who said, “*Do more of this type of partnership. In fact, I have, since this study, established collaborations with other teams of a similar spectrum of skills and roles*.”

### Perceived sustainability

Both groups reported that they would work with their partner again in the future (researchers = 117, 92.9%; knowledge-users = 54, 91.5%). They also believed that considerable time and effort was required to foster and maintain partnerships of this sort but that it was worth it:

“*… I would not trade this experience for anything. It was incredibly worthwhile, and I have made some lifelong friendships in the process. The personal benefits should not be forgotten in this*.” (Researcher).

Several knowledge-users also said that they would not change anything in the partnership, “*it has unfolded in the way* [originally] *anticipated and has even resulted in this partnership being continued in another funded grant that I am leading*.” Another knowledge-user commented, “[The partnership] *has been the first phase of an ongoing project* [because it] *gives an important platform to proceed.*” This sentiment was not shared by all participants. Some knowledge-users reported wanting more time and funding (much like the researchers) as well as more involvement of “*all partners*” and agreement on deliverables.

The survey asked participants to rank strategies that granting agencies could use to facilitate and support partnership sustainability. Participants ranked the six provided options from 1 to 6, with 1 being the most helpful (Tables 4 and 5). While both researchers (*n* = 81, 64.8%) and knowledge-users (*n* = 31, 50%) reported that more time and more resources would be the most helpful, researchers rated this factor to be significantly more important than knowledge-users (*U* = 3166, *P* = 0.021, *r* = − 0.15). Another high-ranking factor in support of sustaining the partnership was having granting agencies share practical information with researchers and knowledge-users about how to successfully apply for grants. A matchmaking service was also provided as an option to support partnership sustainability; knowledge-users had a neutral perspective and researchers thought this would be the least helpful. The rest of the potential options had an equal mix of responses but no other preferences were revealed. There were a few that chose “*Other*” as the most helpful and reported ideas such as “*training programs with KT modules*”, “*track record of working together*” and “*targeted programs in health care research*”.

**Table 4:** Ranked factors that could help sustain partnerships

Researchers	Rank 1	Rank 2	Rank 3	Rank 4	Rank 5	Rank 6	Total
Information for researchers and knowledge-users about how to successfully apply for grants	11 (9.4%)	32 (27.4%)	29 (24.8%)	21 (17.9%)	17 (14.5%)	7 (6%)	117
Workshops or training modules for researchers and knowledge-users to increase relevant skills	8 (6.5%)	14 (11.4%)	33 (26.8%)	33 (26.8%)	30 (24.4%)	5 (4.1%)	123
A matchmaking service for researchers and knowledge-users (so they can find each other)	9 (7.3%)	16 (12.9%)	23 (18.5%)	25 (20.2%)	38 (36.6%)	13 (10.5%)	124
More time (longer grants) and/or more money to conduct this type of research	81 (64.8%)	20 (16%)	12 (9.6%)	7 (5.6%)	3 (2.4%)	2 (1.6%)	125
Staff to work with funded teams to facilitate knowledge translation	12 (9.4%)	38 (29.7%)	17 (13.3%)	27 (21.1%)	29 (22.7%)	5 (3.9%)	128
Other	6 (12%)	2 (4%)	4 (8%)	3 (6%)	1 (2%)	34 (68%)	50

**Table 5:** Ranked factors that could help sustain partnerships

Knowledge-users	Rank 1	Rank 2	Rank 3	Rank 4	Rank 5	Rank 6	Total
Information for researchers and knowledge-users about how to successfully apply for grants	7 (11.7%)	13 (21.7%)	13 (21.7%)	9 (15%)	16 (26.7%)	2 (3.3%)	60
Workshops or training modules for researchers and knowledge-users to increase relevant skills	9 (15.3%)	13 (22%)	11 (18.6%)	17 (28.8%)	8 (13.6%)	1 (1.7%)	59
A matchmaking service for researchers and knowledge-users (so they can find each other)	3 (5.1%)	11 (18.6%)	18 (30.5%)	6 (10.2%)	15 (25.4%)	6 (10.2%)	59
More time (longer grants) and/or more money to conduct this type of research	31 (50%)	9 (14.5%)	8 (12.9%)	10 (16.1%)	3 (4.8%)	1 (1.6%)	62
Staff to work with funded teams to facilitate knowledge translation	8 (12.5%)	16 (25%)	11 (17.2%)	14 (21.9%)	12 (18.8%)	3 (4.7%)	64
Other	4 (16.7%)	2 (8.3%)	1 (4.2%)	0	2 (8.3%)	15 (62.5%)	24

When participants were asked in an open-ended format what they might do differently to support the partnership process, many stated that they would make no changes and considered it a positive experience overall with favourable outcomes. Both groups agreed that more time and money are needed, specifically to be allocated to creating and maintaining the partnerships and to allow for more frequent in-person meetings. Researchers were more likely to suggest formalising and structuring the partnership process from start to finish. Both groups believed that getting clear expectations early in the development stages of the project would foster more sustainable partnerships. Similarly, both knowledge-users and researchers said it would be beneficial to enhance accountability amongst researchers, knowledge-users and the granting agency with frequent check-ins to ensure expectations were being met and miscommunication minimised.

## Discussion

Partnerships with researchers and knowledge-users are expanding because they are understood to be essential to creating impactful research. Although the exact mechanisms to support IKT within different contexts remain unknown [18], the literature does favour the use of IKT for several reasons, including improved research process and uptake of results [16]. We believe that learning from funded researcher–knowledge-user partnerships can help us develop a better understanding of the factors (or mechanisms) that can improve, or support, these types of collaborations in the future. We also believe that the lessons learned through our study can help strengthen rapport between researchers and knowledge-users by calling to light perceived barriers, with the aim to enhance future partnerships.

In our study, the majority of both researchers and knowledge-users reported benefitting from the formally funded partnership and believed their research findings had a greater likelihood of impact. Further, our results echo recent literature demonstrating the benefits of this type of targeted grant funding that supports research co-production or IKT [26]. Our research also identified that longer grant periods and more money to conduct research are highly favoured. Strong partnerships allow research to be launched quickly to support rapid responses to practice-based research questions. Participants identified “*dedicated grant support staff*” as helpful in supporting the access to partnerships. The importance of collaboration, shared vision and trust along with adequate funding and dedicated staff have been previously reported as important IKT mechanisms [16].

Research shows knowledge brokers, as a dedicated role for ensuring the exchange of evidence into practice, can be very effective in supporting KT [27] and play a critical role in supporting partnered grants as IKT facilitators [28]. Providing dedicated support needs to be balanced against the infrastructure needs and capacity-building expectations of the partnership [29].

We also discovered that, although barriers in the partnership existed, they did not seem to impede the project or partnership. This could be the result of several factors. Research has shown that partnerships built on existing relationships are more likely to succeed [30] and existing relationships support the development of practical research questions with feasible solutions [31]. It is possible that most of our participants were successful at obtaining their grant because of an existing relationship. It is important for granting agencies to consider how best to support the development of new partnerships and which mechanisms can be supported or should be present a priori. For example, grant agencies could provide funding opportunities that allow researchers and knowledge-users to meet face-to-face and support initial planning sessions. Many granting agencies, such as CIHR, offer support for these activities through planning grants. These opportunities allow the partners to get to know one another and develop trust. Trust and role clarity in early stages foster better collaboration and co-created research that meets the needs of both parties. However, researchers and knowledge-users may be unaware of grants available for planning purposes. As such, we recommend that granting agencies better promote planning grants to enhance partnership building.

Supportive training environments are often a requirement of funding and can foster skill development in researchers and trainees [32]. While the same can be true for capacity-building in knowledge-users, this requires a large focus on building relationships and participating in IKT, which is often less valued in the traditional academic sense. Conversely, researchers need to be aware of the ‘political cycle’ and how policies (organisational or governmental) shape research needs. The timing, or scheduling, has been acknowledged as a key barrier to successful IKT [33]; equally as important is the development or a nurturing of a research culture among organisations [34]. IKT has been demonstrated as an effective mechanism to improve the uptake of research policy [17]. IKT will be more successful when knowledge-users have a more supportive environment and organisational context that values research as part of its mandate [35].

The degree to which researchers and knowledge-users report being eager to continue these partnerships supports the idea that they perceive value in having multiple perspectives and collaboration in research. A high proportion of researchers and knowledge-users in our study (>90%) reported their intention to continue partnered research in the future. Maintaining a partnership brings new challenges and considerations; while our participants seemed aware of some of these (e.g. staff turnover and timelines), others (such as conflict management) may need to be reconsidered. In our study, very few, if any, participants reported on conflict management strategies.

Successful partnerships thrive on rapport and cooperation [13]. While the disproportionate involvement of researchers and knowledge-users at different stages of the research process may suggest partnerships had variable engagement of partners, it may also point to a more nuanced characteristic of these partnerships. Seasoned researchers may have a better sense of when knowledge-users can most effectively contribute to different phases of the research process. Similarly, knowledge-users are better able to contribute to a project when it is aligned with their expertise; this strengths-based approach to engagement may be key to successful IKT. While our research did not directly explore this, further research could be done to assess the level of engagement at the various stages of a research project to better understand how and when partnerships should be engaged. By taking a ‘strengths-based’ approach to a partnership, partners may feel that they are contributing in an effective and efficacious manner, leading to more satisfaction with the partnership, its outcomes and their overall involvement [36]. More research is needed to understand roles throughout the research process and finding ways to engage knowledge-users in the most efficient and impactful way. This is a challenge, however, as some partnerships require funding to ‘start’ and cannot be ready for a project until the partnership has had time to develop. By taking time to establish norms, trust and role clarity, it is possible that knowledge-users could have a more meaningful role in all stages of the research process and more effectively bridge the gap between research and implementation.

There was an overall belief by participants that the impact of their research was greater because of the partnership. The development of lasting and sustainable partnerships throughout and beyond the research process may not always be possible or needed. Partnerships with knowledge-users should be encouraged differently based both on the stage of the research and the strengths, along with interests of the knowledge-users. Establishing goals and expectations for the various stages of the research may be the most effective approach to ensuring genuine engagement and IKT. Key mechanisms to successful KT requires both parties to be active participants in shaping, conducting and interpreting research to apply findings in practice. This cannot be a ‘one-size-fits-all’ approach. Grants intended to support partnerships should enable both partners to function optimally and thrive in their collaborative efforts throughout the whole project.

### Limitations

Our study has a number of limitations. We developed the survey de novo based on our literature review, which means that we may have missed important concepts or ideas not previously documented in the literature. We were not able to use a previously validated tool because none existed at the time. However, we did pilot the survey to ensure face and content validity. Although the response rates of researchers and knowledge-users were high (81% and 71%, respectively), all participants had been awarded grants from CIHR to conduct collaborative research between 2005 and 2009 and we surveyed them 5–7 years after being funded (2010 to 2012). The findings may therefore not be generalisable to partnered research funded by CIHR more recently; further, they may also not be generalisable to partnered research not funded through CIHR’s IKT programmes (i.e. partnered research supported by other funding agencies in other jurisdictions and countries). As is the case with any self-report survey, there is the potential of bias from the participants [37]. Despite these limitations, we believe that our findings remain relevant as they provide important historical baseline data. This survey was, what we believe to be, the first attempt to systematically understand researcher–knowledge-user experiences and impact from a large cohort of IKT grants funded by a national health research funding agency.

## Conclusion

Partnered research has become more prevalent in and often a requirement of research grants. The goal of this study was to gain a better understanding of funder-required research partnerships and how they influence the research process as well as report on lessons learned to support current granting organisations wanting to use partnered research programmes. In addition, we sought to understand perceived barriers in partnership, how partners overcame these obstacles and intentions for future partnered research. Our findings suggest that, despite barriers and the sometimes less than ideal outcomes, there are great benefits to partnered research that are felt by both researchers and knowledge-users. Our results provide evidence that funding schemes that support knowledge-user and researcher partnerships are worthwhile from the perspective of knowledge-users and researchers. Our findings may also be useful in guiding future studies and collaborative efforts in research to augment partnered research and the accountability of researchers and knowledge-users. Further research should expand on this by identifying factors that support partnership building and sustainability as well as looking more directly at outcomes of partnered research. There is also a need to better understand the quality of partnerships, how some partnerships lead to feelings of equality while others do not, and how research funders can optimally support research partnerships to provide maximal benefits.

## Data Availability

The datasets generated and/or analysed during the current study are not publicly available.

## Abbreviations

CAD: Canadian dollar
CIHR: Canadian Institutes of Health Research
IKT: integrated knowledge translation
KS: Knowledge Synthesis
KT: knowledge translation
KTA: Knowledge to Action
PHSI: Partnerships for Health System Improvement
